# An Extended dataset of occurrences of species listed in Resolution 6 of the Bern Convention from Ukraine

**DOI:** 10.3897/BDJ.10.e84002

**Published:** 2022-06-08

**Authors:** Oleksii Vasyliuk, Oleh Prylutskyi, Oleksii Marushchak, Anna Kuzemko, Iuliia Kutsokon, Oksana Nekrasova, Niels Raes, Mikhail Rusin

**Affiliations:** 1 I.I. Schmalhausen Institute of Zoology of National Academy of Sciences of Ukraine, Kyiv, Ukraine I.I. Schmalhausen Institute of Zoology of National Academy of Sciences of Ukraine Kyiv Ukraine; 2 V. N. Karazin Kharkiv National University, Kharkiv, Ukraine V. N. Karazin Kharkiv National University Kharkiv Ukraine; 3 M.G.Kholodny Institute of Botany of National Academy of Sciences of Ukraine, Kyiv, Ukraine M.G.Kholodny Institute of Botany of National Academy of Sciences of Ukraine Kyiv Ukraine; 4 Netherlands Biodiversity Information Facility (NLBIF), Leiden, Netherlands Netherlands Biodiversity Information Facility (NLBIF) Leiden Netherlands; 5 Naturalis Biodiversity Center, Leiden, Netherlands Naturalis Biodiversity Center Leiden Netherlands; 6 Kyiv Zoo, Kyiv, Ukraine Kyiv Zoo Kyiv Ukraine

**Keywords:** occurrence, Emerald Network, Bern Convention, Red Data Book of Ukraine, rare species, conservation

## Abstract

**Background:**

The dataset includes georeferenced occurrences of species listed in Annex I of Resolution 6 of the Bern Convention and, partly, in the Red Data Book of Ukraine. The dataset was compiled within the work of NGO "Ukrainian Nature Conservation Group" aimed to prepare a Shadow list of Emerald Network (European network Areas of Special Conservation Interest) in Ukraine - newly proposed territories aimed at conservation of particular species and habitats mentioned in Resolution 4 and 6 of the Bern Convention. The list was prepared in 2017-2020 for expanding the already existing Emerald Network of Ukraine. Based on actual registrations of flora and fauna collected and gathered by scientists and naturalists in a form of dataset, which is described in the following paper.

**New information:**

This dataset provides information about 29,938 occurrences of species from the territory of Ukraine listed in Annex I of Resolution 6 of the Bern Convention, as well as in the Red Data Book of Ukraine. This is the largest public dataset on occurrences of rare and endangered species from Ukraine till now. Data presented here laid the foundations for the proposal of 106 approved Emerald Network sites (2019), as well as for 148 Emerald Network sites that were nominated in 2020. New insights on the endemic species *Centaureapseudoleucolepis* Kleopow is provided, which was previously considered to be extinct, according to the IUCN Red List.

## Introduction

The main purpose of the current dataset is to provide free and open access to the data on occurrences of the species listed in Resolution 6 of the Bern Convention (Council of Europe 1998) from Ukraine, extended with data on occurrences of species listed in the 3rd edition of the Red Data Book of Ukraine (Akimov 2009, Didukh 2009). The dataset comprises both processed published data and personal observations, with a significant predominance of published data that make up 95% of the dataset. The dataset has resulted from efforts on biodiversity data mobilisation for development of the Emerald network in Ukraine (Polyanska et al. 2017).

The compilation of this dataset began in 2007; since then, it has been continuously updated, based on results of recent field research. From 2007 to 2015, a group of Ukrainian biologists founded an informal group “Save Ukrainian steppes!”, which aimed to prevent forest planting in the steppe zone of Ukraine, in places of natural dry grassland habitats. Amongst other activities, the group began collecting information on the findings of steppe species protected by the Red Data Book of Ukraine.

Since 2016, group members launched a new initiative in Ukraine – expanded territories of the Emerald Network in Ukraine. The first version of the Emerald Network in Ukraine was developed without taking into account data on the distribution of species listed in Resolution 6 and in the habitats listed in Resolution 4 of the Bern Convention (hereinafter BC), but simply included existing large protected areas. The Emerald Network sites at that time almost did not cover steppe ecosystems. The authors of this article, in collaboration with other scientists, started to gather information on the distribution of species listed in Resolution 6 of the BC. We have combined the collection of new data with the existing dataset because the distribution of rare steppe species fully corresponds with the distribution of habitat types E1.2 and E1.13 of Resolution 4 of the BC. Based on these data, we prepared justifications for 106 Emerald Network sites (1.4 million ha), which were approved by the Bern Convention in December 2019 and for an additional 148 sites (2.7 million ha), which are currently under consideration (Kuzemko et al. 2020).

There were relatively few publications providing exact taxon occurrence data in Ukraine before 2017. Except for the collection of papers under the general title “Records of Animals from the Red Data Book of Ukraine”, containing 1400 records (Kostiushyn 2008), most of the historical publications avoided providing precise information about location and time of species observations. Good exceptions were ornithological journals that published such detailed observations, including “Branta: Collection of scientific works of the Azov-Black Sea Ornithological Station” (ISSN 1994-1722, 1430 published records), “Avifauna of Ukraine” (ISSN 1727-7531, 326 records) and “Berkut” (ISSN 1727-0200, 1770 records), “Nature reserves in Ukraine” (ISSN 1729-7184, 929 records) and “Troglodytes” (ISBN 978-966-397-149-7, 161 records). Some regional collections also promoted publication of precise observations, for example, “Birds of the basin of the Siverskyi Donets” (Kharkiv, 395 published records), “Proceedings of II – IV conferences of young ornithologists of Ukraine” (Chernivtsi, 256 records) and “Nature reserves of Crimea” (Simferopol) (226 records). We also processed the personal publication archives of Y. Kutsokon, O. Shynder, M. Peregrym, G. Goncharov, A. Kuzemko and I. Moysiyenko, as well as other researchers who actively published biodiversity data.

In 2008 – 2012, one of the first Ukrainian citizen science projects “Involving the public into biodiversity monitoring” was carried out, for which we created a special webpage Biomon.org (archived copy) for submitting individual amateurs’ observations of three pilot species – *Lucanuscervus* L. (527 observations), *Papiliomachaon* L. (288 observations) and *Liliummartagon* L. (16 observations). Additionally, in 2010, we involved young naturalists club members in data harvesting. We established a connection with the most active groups of school naturalists from Eastern Ukraine, from which we received data about 931 records of rare species of plants. Another valuable contribution was the publication of the “Records of plants listed in the Red Data Book of Ukraine in the Starobilsky steppe” (Butkova 2019), with 740 records, comprising results of rare plant species mapped across Luhansk Region for over 20 years. For citizen science projects, we double-checked all records, based on the photos (species identification accuracy) and satellite imageries with locations of each record manually drawn (georeferencing accuracy), provided by pupil's and young naturalist's club heads.

Another important source of data was regional conservation publications, such as the “Red Data Books” of Donetsk (Averchuk et al. 2010, 1921 records), Dnipropetrovsk (Travleev 2010, 683 records), Luhansk (Maslova et al. 2003, 221 records) oblasts, of Priazovsky Region (Ostapko and Kolomiychuk 2012, 151 records). Additionally, the valuable source of data on species occurrences were monographs (Tarashchuk et al. 1997, Ostapko 2001, Shelegeda and Shelegeda 2001, Melnyk and Parubok 2004, Tishchenko 2006, Kolomiyets et al. 2008, Sova 2011, Kolomiychuk et al. 2012, Matviichuk et al. 2015).

Most of the processed literature sources are hardly accessible. They have been generally available only as hard copies, were not present on the Web and published in Ukrainian or Russian exclusively. Most are so-called “grey literature” – proceedings of local conferences and non-periodical paper collections of protected areas and are not indexed by any database. However, such “grey” editions often accumulate valuable biodiversity data, because most of Ukrainian peer-reviewed journals did not accept data papers until quite recently.

To facilitate data harvesting, we initiated publishing of data paper collections (Akimov et al. 2018a, Akimov et al. 2018b, Akimov et al. 2019, Kuzemko 2019, Rusin and Ghazali 2019, Kuzemko et al. 2020, Rusin et al. 2021), which were further digitised and published as separate GBIF datasets (Marushchak et al. 2020, Babiichuk et al. 2021, Vasyliuk et al. 2021, Kharchenko et al. 2021, Rusin et al. 2021). Since 2018, we issued seven such collections, which included 58,478 new records of rare species of animals, plants and fungi from Ukraine.

The materials of the present dataset have been widely used for conservation purposes. For example, about 60 protected areas were created in Ukraine using this information (Kuzemko et al. 2020). We also published four popular publications on the distribution of rare plant species in certain regions of Ukraine (Peregrym et al. 2021). During 2021, over 50 environmental impact assessment acts were corrected or revoked considering data on protected species distribution from the present dataset.

## Project description

### Title

Mobilisation of biodiversity data from Ukraine to GBIF

### Personnel

Mikhail Rusin, Svetlana Miteva

### Study area description

Ukraine

### Funding

The dataset has been organised with the support of Project nlbif2018.2019.004, funded by NLBIF to The Habitat Foundation “Mobilization of biodiversity data from Ukraine to GBIF” https://www.nlbif.nl/mobilization-of-biodiversity-data-from-ukraine-to-gbif/

## Sampling methods

### Study extent

Ukraine, all territory

### Sampling description

For literature occurrences, we selected only observations which met the following conditions:

- species listed in Annex I of Resolution 6 of the Bern Convention (Council of Europe 1998) and/or Red Data Book of Ukraine (3rd ed., Akimov 2009);
- observation accompanied by coordinates or precise locality description;
- source of identification is reliable (either reported by professional researchers or accompanied by high-quality photos for citizen-science observations).

### Quality control

Data were double-checked in terms of identification and georeferencing accuracy.

- For citizen science projects, we double-checked all records, based on the photos (species identification accuracy) and satellite imageries with locations of each record manually drawn (georeferencing accuracy), provided by pupil's and young naturalist's club heads along with the data.
- Species identification extracted from peer-reviewed scientific publication were taken as is, but checked for name misspelling against GBIF Species Matching tool.
- Coordinates of records were double-checked visually using Google Earth service (Google 2021).

### Step description

The dataset comprises both published data and personal observations. The following steps were taken:

1. Selection of reliable literature resources on species occurrences in the territory of Ukraine (total 641 sources);
2. Manual data extraction from published taxonomic treatments (species-date-place);
3. Manual georeferencing of records based on descriptions of the localities using Google Maps (Google 2021);
4. Aggregating and quality checking of citizen-science observations;
5. Aggregating personal observations of the authors;
6. Data post-processing using Darwin Core terms (Wieczorek et al. 2012);
7. Data cleaning using OpenRefine (OpenRefine 2022).

## Geographic coverage

### Description

The entire territory of Ukraine.

**Figure 1.**
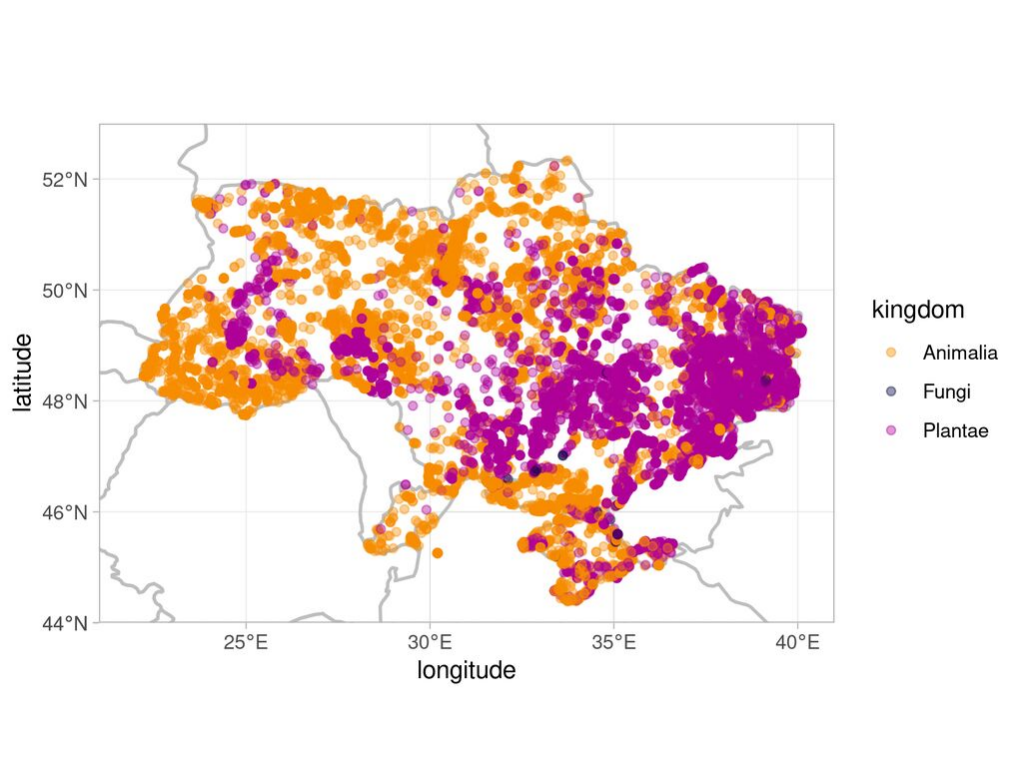
Distribution map of the occurrences from the extended dataset of registration points of species listed in Resolution 6 of the Bern Convention. There is some sampling bias towards steppe zone in plant records, due to extensive plant data mobilisation in those regions for Emerald Network development purposes.

Occurrences of plant species tend to be more frequent towards the steppe (semi-arid and arid) zone of the country, while animal observation records are more evenly distributed across the territory of Ukraine (Fig. 1). Revealing of true spatial distribution of Ukrainian biodiversity was not an aim of the present paper and we are aware that this spatial pattern reflects some sampling bias towards steppe ecosystems. The main reason is that the steppe ecosystems, despite being vulnerable, have been less represented in current protected area network than ecosystems of forested regions, including Carpathians (Vasyliuk et al. 2021b). Based on the existing estimation (Parnikoza and Vasyliuk 2011), only 3% of historical Ukrainian steppes remains; therefore, more efforts have been made to study those territories. Since data collected during the project aimed to develop the Emerald Network in Ukraine were a significant part of the dataset, it may cause a sampling bias towards steppe plant data. Fungal records are sparse.

### Coordinates

44.277 and 52.376 Latitude; 21.884 and 40.254 Longitude.

## Taxonomic coverage

### Description

**Figure 2.**
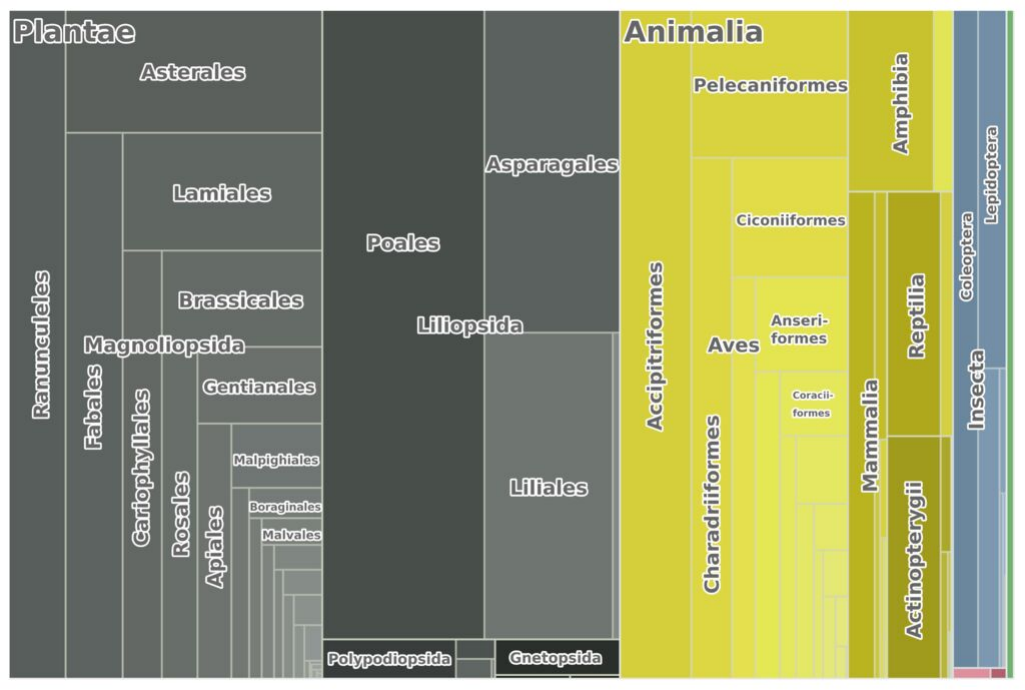
Taxonomic distribution of occurrences from the extended dataset of registration points of species listed in Resolution 6 of the Bern Convention.

The dataset contains 18,553 occurrences of plants, 11,197 occurrences of animals and 188 occurrences of fungi (Marushchak et al. 2022). The latter are not listed in Annex I of the Bern Convention yet, but are included in the Red Data Book of Ukraine (Fig. 2). Prevalent taxa of plants were vascular plants, while prevalent animal taxa were vertebrates.

### Taxa included

**Table:** 

Rank	Scientific Name	
kingdom	Plantae	
kingdom	Animalia	
kingdom	Fungi	

## Temporal coverage

**Data range:** 1903-1-01 – 2020-12-31.

### Notes

**Figure 3.**
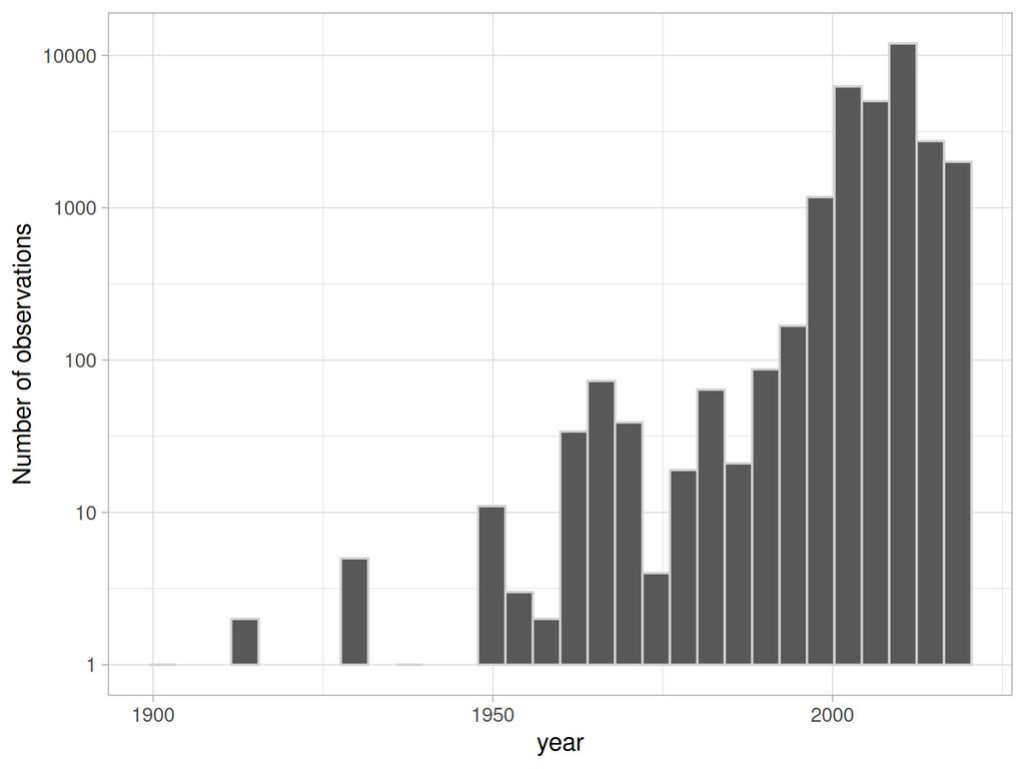
Temporal distribution of records. For the records without exact date of observation, mean dates were calculated, based on the known time window. Records which time windows exceeded 10 years were excluded (228 records). Y-axis is log-scaled.

Most of the observations (28,147, 94%) were made since 2000. (Fig. 3).

## Usage licence

### Usage licence

Open Data Commons Attribution License

## Data resources

### Data package title

An extended dataset of registration points of species listed in Resolution 6 and 4 of the Bern Convention.

### Resource link

https://www.gbif.org/dataset/20f7dc5e-f6b6-4cba-8db3-4a522b7d08d8

### Alternative identifiers

https://doi.org/10.15468/hmd8az

### Number of data sets

1

### Data set 1.

#### Data set name

An extended dataset of registration points of species listed in Resolution 6 and 4 of the Bern Convention.

#### Data format

Darwin Core

#### Description

The dataset includes a tabulation-delimited table with 26 fields in Darwin Core terms and 29,938 records in it.

**Data set 1.** 

Column label	Column description
occurrenceID	https://dwc.tdwg.org/terms/#dwc:occurrenceID; an identifier of a particular occurrence, unique within this dataset. Since the data were collected within different data mobilisation projects, we used a combination of the project’s abbreviation and incremental numbers.
scientificName	https://dwc.tdwg.org/terms/#dwc:scientificName; the original names as provided in publication, but corrected for spelling mistakes using GBIF Species Matching tool. Some species which are not covered by GBIF Backbone taxonomy yet treated according to original spelling in the data source.
kingdom	https://dwc.tdwg.org/terms/#dwc:kingdom; The full scientific name of the kingdom in which the taxon is classified.
phylum	https://dwc.tdwg.org/terms/#dwc:phylum; The full scientific name of the phylum or division in which the taxon is classified.
class	https://dwc.tdwg.org/terms/#dwc:class; The full scientific name of the class in which the taxon is classified.
order	https://dwc.tdwg.org/terms/#dwc:order; The full scientific name of the order in which the taxon is classified.
family	https://dwc.tdwg.org/terms/#dwc:family; The full scientific name of the family in which the taxon is classified.
genus	https://dwc.tdwg.org/terms/#dwc:genus; The full scientific name of the genus in which the taxon is classified.
specificEpithet	https://dwc.tdwg.org/terms/#dwc:specificEpithet; The name of the first or species epithet of the scientificName.
taxonRank	https://dwc.tdwg.org/terms/#dwc:taxonRank; The taxonomic rank of the most specific name in the scientificName.
eventDate	https://dwc.tdwg.org/terms/#dwc:eventDate; the full date of the observation as it might be extracted from the publication. In some cases – as date ranges. Many sources we used did not contain precise information about the date of each observation, only the overall research time-window. Since intervals cannot be reduced to any particular date, GBIF.org automatically downscales such intervals to the 1st Jan of the first Year of the time-window, which may be misleading for the people referenced directly to the GBIF.org portal. Please download our data as DarwinCore archive, which contains dates as input. Any user’s download of those data, including search query results, will return true dates. Moreover, GBIF.org displays full information from all the fields for each record by clicking on it (as an example: https://www.gbif.org/occurrence/3014589991).
basisOfRecord	https://dwc.tdwg.org/terms/#dwc:basisOfRecord; the method in which data were acquired. Two levels: "HumanObservation" for authors' observations and personal communications, "MaterialCitation" for occurrences derived from scholarly publications.
decimalLatitude	http://rs.tdwg.org/dwc/terms/decimalLatitude; The geographic latitude in decimal degrees.
decimalLongitude	https://dwc.tdwg.org/terms/#dwc:decimalLongitude; The geographic longitude in decimal degrees.
coordinateUncertaintyInMeters	https://dwc.tdwg.org/terms/#dwc:coordinateUncertaintyInMeters; the distance (in metres) from the given decimalLatitude and decimalLongitude describing the smallest circle containing the whole of the Location. Set as 50 m for GPS coordinates and 1000 m for the coordinates georeferenced, based on description.
geodeticDatum	https://dwc.tdwg.org/terms/#dwciri:geodeticDatum; the geodetic datum upon which the geographic coordinates are given. All values are WGS84.
georeferencedBy	https://dwc.tdwg.org/terms/#dwc:georeferencedBy; a person who determined the georeference.
georeferenceProtocol	https://dwc.tdwg.org/terms/#dwciri:georeferenceProtocol; A description of the method used to determine coordinates.
recordedBy	https://dwc.tdwg.org/terms/#dwc:recordedBy; A person or group of people who determined the georeference for the Location.
organismQuantityType	https://dwc.tdwg.org/terms/#dwciri:organismQuantityType; The type of quantification system used for the quantity of organisms. “Individuals” for the most of occurrences, but in some cases also “pairs” for birds and projected coverage for plants were used.
organismQuantity	https://dwc.tdwg.org/terms/#dwc:organismQuantity; A number or enumeration value for the quantity of organisms, according to the values in the organismQuantityType field.
language	https://dwc.tdwg.org/terms/#dc:language ; A language of the resource. One value - en | uk, because each observation combined fields both in English and Ukrainian.
associatedReferences	https://dwc.tdwg.org/terms/#dwc:associatedReferences; bibliographic references, datasets and data collection project name associated with the Occurrence.
countryCode	https://dwc.tdwg.org/terms/#dwc:countryCode; one value – UA.
country	https://dwc.tdwg.org/terms/#dwc:country; one value – Ukraine.
stateProvince	https://dwc.tdwg.org/terms/#dwc:stateProvince; The name of the administrative region of Ukraine in which the Location occurs (name of the administrative region (Oblast’) or Autonomous Republic of Crimea or Kyiv City).
municipality	https://dwc.tdwg.org/terms/#dwc:municipality; The full name of the next smaller administrative region than Oblast’ (region of the Ukraine) in which the Location occurs. Official administrative division of Ukraine of 2020 was used.
verbatimLocality	https://dwc.tdwg.org/terms/#dwc:verbatimLocality; verbal description of the locality as was provided by the authors of observations (in Ukrainian).

## Additional information

### Conservation status of the species

The dataset comprises information on occurrences of the 384 species that are assigned to one of the IUCN conservation categories (IUCN Standards and Petitions Committee 2022, Fig. 4). One species – *Centaureapseudoleucolepis* Kleopow, which inhabits rocky areas (granite outcrops) – is currently considered Extinct, according to IUCN (Melnyk 2011).

This species is endemic to Ukraine (Buord and Lesouëf 2006) and is accepted at the species level in some generally accepted nomenclature sources, for example, Euro+Med (2006). It is reported that this species has last been seen in the 1930s and has not been found since then, despite extensive surveys (Melnyk 2011). However, this information is not relevant, as the locality of this species has been repeatedly confirmed in recent decades in "Kamiani Mohyly", the Ukrainian Steppe Natural Reserve. This statement is proved by two confirmed localities of this species on the citizen science portal 'iNaturalist' by V. Kolomiychuk (link) and G. Boyko (link), both of the research grade and available at GBIF.org (GBIF.org 2022). Taking into analysis occurrences of *C.pseudoleucolepis* currently available in GBIF (GBIF.org 2022), the species Extent of Occurrence is preliminarily estimated as 26.5 km^2^, while Area of Occupancy as 16 km^2^, which is close to the thresholds for 'Critically Endangered' (CR) and 'Endangered' (EN) categories of the IUCN Red List of Threatened Species (Bachman et al. 2011, IUCN Standards and Petitions Committee 2022). Therefore, we consider that the conservation status of this species requires updating, taking into consideration also possible unpublished records and population trends.

### Other public resources on occurrences of rare and threatened species in Ukraine

Though we attempted to consolidate the vast majority of the data on occurrences of rare and threatened species in Ukraine, it should be mentioned that there are several other continuously updated resources where such an information can be found. For example, a significant amount of data is stored by both global biodiversity data mobilisation projects like iNaturalist (link) and eBird (link) and local citizen-science initiatives like UkrBIN (link), though the first two are also available through the GBIF network.

## Figures and Tables

**Figure 4.**
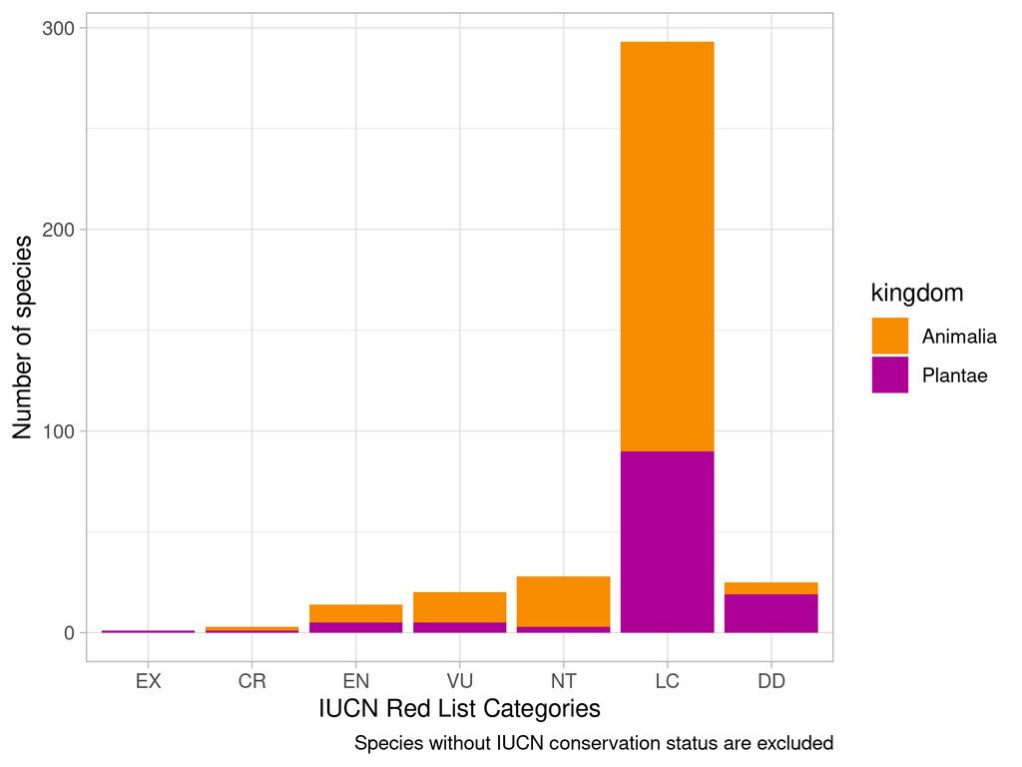
IUCN conservation status for evaluated species.
